# Diagnostic Accuracy of Artificial Intelligence for Detection of Meniscus Pathology on Magnetic Resonance Imaging: A Systematic Review

**DOI:** 10.7759/cureus.91832

**Published:** 2025-09-08

**Authors:** Peter A Giammanco, Christopher E Collins, Sunny M Trivedi, Reem O Sarsour, Mikayla Kricfalusi, Joseph G Elsissy

**Affiliations:** 1 Orthopedic Surgery, Arrowhead Regional Medical Center, Colton, USA; 2 Orthopedic Surgery, California University of Science and Medicine, Colton, USA; 3 Orthopedic Surgery, Loma Linda University Medical Center, Loma Linda, USA; 4 School of Medicine, California University of Science and Medicine, Colton, USA

**Keywords:** artificial intelligence, machine learning, magnetic resonance imaging, meniscus, meniscus tear

## Abstract

Meniscus tears can be difficult to diagnose on magnetic resonance imaging (MRI) due to the various types of tears. As artificial intelligence (AI) continues to advance, it could serve as a valuable tool that assists physicians with diagnostic accuracy and efficiency. The purpose of this study was to analyze the performance of AI in diagnosing meniscus tears on MRI using an array of studies and to compare the performance of AI to that of physicians.

A literature search was conducted on PubMed and Embase for articles regarding the use of AI for the detection of meniscus tears. AI model, number of MRI studies, sensitivity, specificity, accuracy, AUC, and comparison to physicians encompassed the data that was extracted.

A total of 18 studies, comprising 47,621 MRIs, were included in this review. The AUC of these AI models ranged from 0.781 to 0.984, averaging 0.912±0.053. The pooled sensitivity and specificity were 0.831±0.097 and 0.868±0.088, respectively. Three of these studies compared their results to those of 10 radiologists and two orthopedic surgeons. When comparing the sensitivity and specificity of AI models to those of physicians, Cochrane’s Q was statistically significant (p < 0.01) with large heterogeneity amongst the two groups (I^2^ = 84.72%).

The results suggest that AI’s ability to detect meniscus lesions on MRI was relatively strong. When this performance was compared to that of physicians, the results were comparable, highlighting the potential benefits in a clinical setting.

## Introduction and background

Meniscus tears are a relatively common intra-articular knee injury that occurs across an array of patient demographics, from young, physically active adolescents to professional athletes to amateur, middle-aged adults. It has been reported that meniscus lesions account for about 32% of all internal knee injuries [1]. This may be due, in part, to the rising popularity of recreational sports that require substantial torsional activity from the knee joint, such as soccer, basketball, pickleball, and snow sports, as approximately 25% of all knee injuries occur during sport participation [1]. The estimated incidence of meniscus injury is 0.61 to 0.70 per 1000 person-years in the United States, and it has been estimated that approximately 23% of these injuries require surgical intervention [1,2].

Magnetic resonance imaging (MRI) is widely recognized as the imaging of choice when there is concern for meniscus pathology due to its ability to image soft tissue injuries and detect fluid collections across multiple anatomical planes with high resolution [3]. Properly identifying meniscus lesions is important because they are the single most common reason for knee arthroscopy [4]. However, the accuracy of MRI with regard to diagnosing meniscus pathology has been questioned. The most recent reports on the accuracy of radiologists in detecting medial and lateral meniscus tears measure 77.5% and 85.8%, respectively, when compared against arthroscopically confirmed positive meniscus cases [5]. This may be a result of the various types of meniscus tears or the notion that radiologists experience and image quality vary across institutions [6,7]. Additionally, it is important to note that there is a shortage of highly trained musculoskeletal radiologists, which also contributes to long waiting times for results [7,8]. Ultimately, as physicians regularly order MRIs for possible meniscal pathology, the cost-effectiveness of these MRIs comes into consideration [9]. Thus, there may be a role for artificial intelligence (AI) models to be a potential solution to these concerns regarding MRI, as AI has already been shown to improve accuracy and efficiency on other types of radiologic imaging [10].

AI algorithms, such as Convolutional Neural Networks (CNN), go through a training phase in which they are given MRI images with and without meniscus tears confirmed via a radiologist’s report or arthroscopy. The AI model then breaks the complex images into different layers and extracts features from each layer to determine the relationship patterns between normal and pathologic. The AI model can then use pattern recognition on unseen imaging in the decision-making process to generate reads with accuracy and efficiency [11]. Extensions of these algorithms, such as cascading U-net, unsupervised data augmentation (UDA), and visual gain information features (VIGF), are techniques that then build on the foundational architecture of CNNs to further enhance segmentation, feature extraction, or identification of a model.

For meniscus pathology, several studies have deployed CNNs, or the likes thereof, to detect meniscus abnormalities and subsequently collected quantitative measurements regarding their performance. Some of these studies have then compared the results of AI to those of orthopedic surgeons and radiologists. There has yet to be a systematic analysis of how the performance of these AI algorithms compares to that of physicians. This study aims to report the current state of AI’s ability to detect meniscus pathology on MRI and to assess the diagnostic performance of AI models in comparison to orthopedic surgeons and radiologists. It was hypothesized that AI models would perform similarly to those of orthopedic surgeons and radiologists.

## Review

Methods

The study was conducted in accordance with Preferred Reporting Items for Systematic Reviews and Meta-Analysis (PRISMA) guidelines. Inclusion criteria consisted of: the use of a deep learning model, such as CNN, or an extension of one, deploying said AI model to detect meniscus pathology, doing so on MRI imaging, and reporting at least one of sensitivity, specificity, accuracy, or AUC. Data extracted from each study for the purposes of diagnostic performance included sensitivity, specificity, accuracy, and AUC, if applicable. If any of these diagnostic performance metrics were missing in the study, the study was still included in this review in order to compile as much data as possible. Exclusion criteria consisted of: combined analysis of meniscus pathology with other knee pathologies, and no reporting of the aforementioned diagnostic measurements. If a study evaluated a physician’s ability to diagnose meniscus pathology on the same images, then these same four metrics were recorded regarding the physician’s diagnostic performance, if applicable. Other data elements extracted included the total number of MRI studies, the type of meniscus pathology, and the AI model. Reported ground truths were either a radiologist’s report or arthroscopic findings.

A systematic search was conducted on two primary databases, PubMed and Embase, using the following string of Medical Subject Headings (MESH) terms: ((mri) OR (“magnetic resonance imaging”)) AND (meniscus) AND ((“artificial intelligence”) OR (“deep learning”) OR (“machine learning”)). Eligible studies were included up until June 2024, and data were collected in a Microsoft Excel sheet. Four researchers screened for eligible studies based on abstracts and titles. Studies were labeled as eligible if they met all four inclusion criteria. If deemed eligible, the full text was evaluated for appropriate data collection. The primary endpoint was statistically significant differences in quantitative measurements (sensitivity and specificity) between artificial intelligence and physicians.

A modified MINORS scoring criterion, which has previously been used in systematic reviews of AI in orthopedics, was applied to the 18 studies that met the inclusion criteria to evaluate risk of bias [12]. The quality was assessed utilizing a binary scale for each criteria, based on the following six metrics: 1: the identification of a clear study aim, 2: description of inclusion and exclusion criteria for the imaging inputs, 3: presence of a ground truth for the images, 4: a report of the distribution of data set, 5: a description of how the AI model performance was assessed, and 6: a description of the AI model used. A maximum score of six reflects high methodological quality, while a minimum score of zero is associated with poor methodological quality. Two independent raters assessed all the studies, and disagreements were resolved by consensus. The inter-observer reliability was excellent at 0.98 (95% Confidence Interval, 0.95-0.99).

Sensitivity, specificity, accuracy, and AUC of AI models were collected for every study, if applicable, and the mean values for these metrics were calculated and reported if studies met the inclusion and exclusion criteria to allow for pooling of data. Secondly, studies that specifically compared AI performance to physicians were evaluated. The mean sensitivity and specificity of physician performance were pooled across studies to produce one pooled sensitivity and specificity for physicians. Similarly, a new pooled average sensitivity and specificity were calculated for the AI models within the subset of studies that compared AI model performance to physicians.

**Figure 1:**
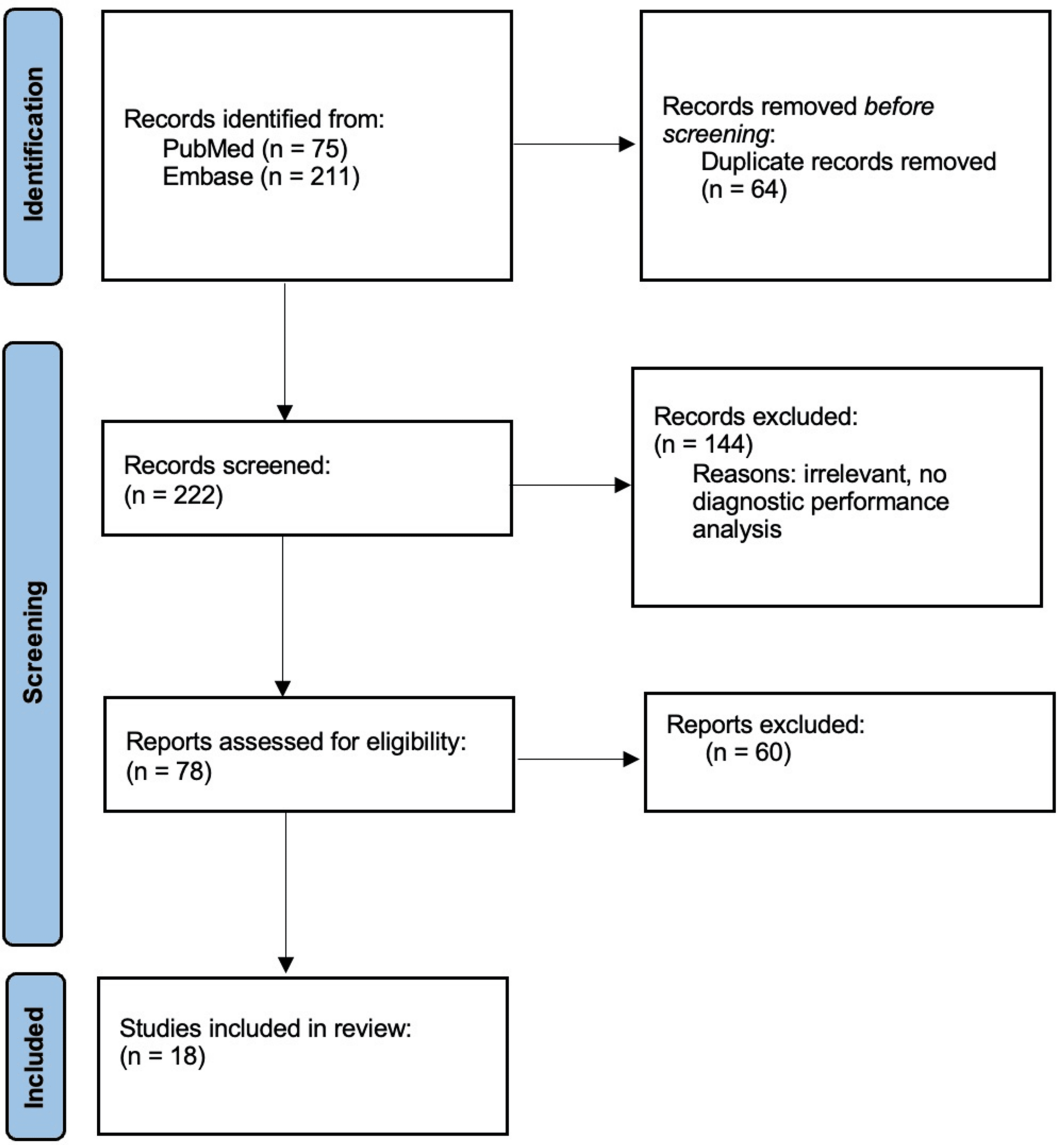
Preferred Reporting Items for Systematic Reviews and Meta-Analyses (PRISMA) Flowchart

To evaluate the overall performance of AI compared to physicians, a composite variable was created. To convert the percentages of each quantitative measurement into binary data, a threshold of 0.8 was used to distinguish upper and lower values of “1” for values equal to or above 0.80 and “0” for values less than 0.80, as depicted in Figure 1. The mean of sensitivity and specificity was used to determine a cutoff value of 0.8. Most studies compared the performance of numerous physicians to one AI model. To generate a 1:1 comparison between AI and physician values and enable statistical evaluation, the difference in sample sizes was addressed by treating “missing” AI values as “0”. In this manner, conservative estimates for the “missing” AI values were utilized, which allowed for direct comparison between the two groups.

A bivariate model that jointly examined sensitivity and specificity was then constructed. The binary composite variable of sensitivity and specificity was utilized. Cochrane’s Q test was used to measure the binary, categorical scores to determine if the proportions of scores were consistent across AI and physician groups. The I2 statistic was calculated as a measure of heterogeneity between AI and physician scoring groups. These statistical analyses were performed using Python (version 3.13.0) by a trained statistician. This study was exempt from IRB approval based on the study design. 

Results

**Figure 2:**
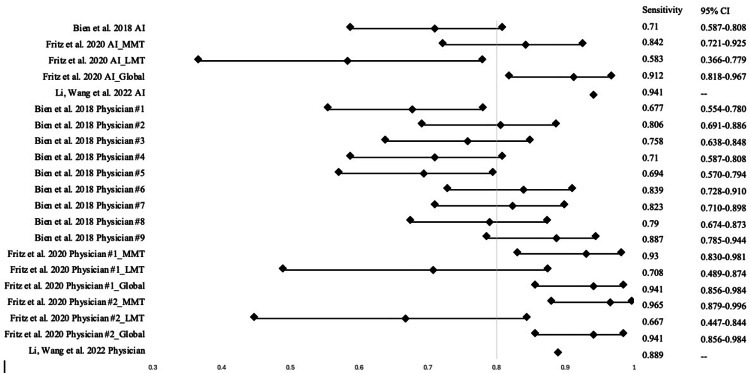
A forest plot depicting the sensitivity scores of AI models and physicians AI: artificial intelligence; MMT: medial meniscus tear; LMT: lateral meniscus tear; Global: global meniscus tear [14, 18, 21]

**Figure 3:**
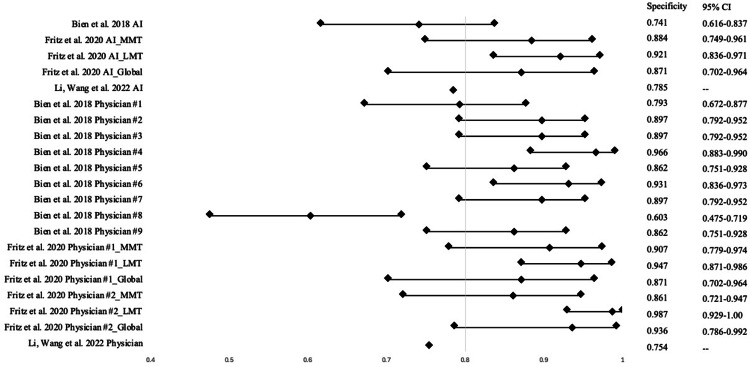
A forest plot depicting specificity scores of AI models and physicians AI: artificial intelligence; MMT: medial meniscus tear; LMT: lateral meniscus tear; Global: global meniscus tear [14, 18, 21]

The database searches retrieved 286 results, 75 from PubMed and 211 from Embase (Figure 1). From there, duplicates and articles that fit the exclusion criteria were removed, leading to 78 studies being screened by the authors. After a thorough screening process, a total of 18 studies, from 2018 to 2024, were included in this systematic analysis, with the total number of MRI studies reaching 47,621 [13-30]. Three of the included studies measured the diagnostic performance of orthopedic surgeons and radiologists on the same images. One study analyzed two radiologists, one study included seven radiologists and two orthopedic surgeons, and the final study evaluated one radiologist. Sensitivity and specificity scores of AI models and physicians from individual studies can be visualized in Figures 2-3, respectively.

The pooled sensitivity, specificity, and accuracy of the AI models were 0.831±0.097 (range, 0.583-0.955), 0.868±0.088 (range, 0.615-0.983), and 0.872±0.062 (range, 0.725-0.970), respectively. The AUC of these artificial intelligence models ranged from 0.781 to 0.984, with an average of 0.912±0.053 (Table 1). The pooled sensitivity, specificity, and accuracy of the physicians were 0.814±0.104 (range, 0.667-0.965), 0.873±0.094 (range: 0.603-0.987), and 0.854±0.069 (range: 0.700-0.940), respectively (Table 2). Of the studies that compared AI model performance to physicians, the pooled sensitivity, specificity, and accuracy of these AI models were 0.798, 0.840, and 0.850 (Table 2). When comparing the performances in sensitivity and specificity of AI models to physicians, Cochrane’s Q was statistically significant (p < 0.01), and I2 revealed large heterogeneity among the two groups (I2 = 84.72%).

**Table 1:** Diagnostic performance of AI algorithms AI: Artificial Intelligence; MMT: Medial Meniscus Tear; LMT: Lateral Meniscus Tear; AM: Anteromedial; AL: Anterolateral; PM: Posteromedial; PL: Posterolateral Data from Xijing_Knee dataset* Data from FastMRI_Knee dataset**

Study	Total MRI Studies	Algorithms Tested	Algorithm Type	Pathology	Sensitivity	Specificity	Accuracy	AUC
Astuto et al. 2021 [13]	1435	1	3D-CNN	Meniscus tear	0.850	0.850	-	0.930
Bien et al. 2018 [14]	1370	1	CNN	Meniscus tear	0.710	0.741	0.725	0.847
Chou et al. 2023 [15]	811	1	CNN	AM meniscus tear	0.850	0.973	0.955	0.973
				AL meniscus tear	0.882	0.983	0.970	0.973
				PM meniscus tear	0.913	0.972	0.962	0.894
				PL meniscus tear	0.848	0.980	0.947	0.984
Couteaux et al. 2019 [16]	1128	1	Mask R-CNN	Meniscus tear	-	-	-	0.906
Dai et al. 2021 [17]	1370	3	CNN	Meniscus tear	0.869	0.800	0.830	0.939
					0.881	0.824	0.848	0.945
					0.879	0.834	0.855	0.954
Fritz et al. 2020 [18]	20,520	1	DCNN	MMT	0.842	0.884	0.860	0.882
				LMT	0.583	0.921	0.840	0.781
				Global meniscus tear	0.912	0.871	0.900	0.961
Hung et al. 2023 [19]	584	1	CNN	Meniscus tear	0.948	0.961	0.954	-
Li et al. 2022 [20]	1104	1	Mask R-CNN	Meniscus tear	0.744	-	0.870	-
Li et al. 2022 [21]	546	1	3D-Mask RCNN	Meniscus tear	0.941	0.785	0.924	0.907
Luo et al. 2024 [22]	4014	1	VIFG	Meniscus injury	-	-	0.850*	-
					-	-	0.863**	-
Mangone et al. 2023 [23]	564	1	CNN	MMT	0.750	0.920	0.837	
Pedoia et al. 2019 [24]	1481	1	3D-CNN	Meniscus lesion	0.898	0.820	-	0.890
Qiu et al. 2021 [25]	205	1	CNN	Meniscus injury	0.914	0.947	0.939	0.968
Rizk et al. 2021 [26]	8058	1	3D-CNN	MMT	0.890	0.840	0.870	0.930
				LMT	0.670	0.880	0.820	0.840
Roblot et al. 2019 [27]	1823	1	CNN	Meniscus tear	-	-	-	0.940
Shin et al. 2022 [28]	1048	1	CNN	MMT	0.832	0.867	0.851	0.889
				LMT	0.680	0.852	0.805	0.817
				MMT and LMT	0.786	0.933	0.920	0.924
Xue et al. 2022 [29]	1242	1	Cascading U-net	Meniscus injury	0.955	0.615	0.832	-
Zhong et al. 2023 [30]	318	1	UDA	Meniscus tear (OA phenotype)	0.750	0.783	0.780	0.900
Total	47621			Average	0.831	0.868	0.872	0.912

**Table 2:** Diagnostic performance of AI compared to physicians AI: artificial intelligence; MMT: medial meniscus tear; LMT: lateral meniscus tear

Study	Pathology	Sensitivity AI	Specificity AI	Accuracy AI	Physician	Sensitivity Physician	Specificity Physician	Accuracy Physician
Bien et al. 2018 [14]	Meniscus tear	0.710	0.741	0.725	Radiologist #1	0.677	0.793	0.733
					Radiologist #2	0.806	0.897	0.850
					Radiologist #3	0.758	0.897	0.825
					Radiologist #4	0.710	0.966	0.833
					Radiologist #5	0.694	0.862	0.775
					Radiologist #6	0.839	0.931	0.883
					Radiologist #7	0.823	0.897	0.858
					Orthopedic Surgeon #1	0.790	0.603	0.700
					Orthopedic Surgeon #2	0.887	0.862	0.875
Fritz et al. 2020 [18]	MMT	0.842	0.884	0.860	Radiologist #1	0.930	0.907	0.920
	LMT	0.583	0.921	0.840		0.708	0.947	0.890
	Global meniscus tear	0.912	0.871	0.900		0.941	0.871	0.920
	MMT				Radiologist #2	0.965	0.861	0.920
	LMT					0.667	0.987	0.910
	Global meniscus tear					0.941	0.936	0.940
Li, Wang et al. 2022 [21]	Meniscus tear	0.941	0.785	0.924	Radiologist #1	0.889	0.754	0.835

The average modified MINORS score was 5.22±0.88 (Table 3), reflecting the relatively high quality of methodology among the studies. The most common reasons for loss of points were failure to describe the inclusion and exclusion criteria for patient and image selection (n=10, 55.6%), followed by failure to define a ground truth (n=3, 16.7%). Eight studies (44.4%) achieved a perfect score of 6, while only one study (5.6%) earned a score of 3 or lower.

**Table 3:** A modified MINORS scoring criteria for risk of bias assessment AI: Artificial Intelligence; 1=yes; 0=no MINORS: Methodological Index for Non-Randomized Studies

Study	Clear Study Aim	Inclusion and Exclusion Criteria	Ground Truth	Report of Distribution of Data Set	Described How AI Performance was Assessed	Described the AI Model Used	Total
Astuto et al. 2021 [13]	1	1	1	1	1	1	6
Bien et al. 2018 [14]	1	0	1	1	1	1	5
Chou et al. 2023 [15]	1	1	1	1	1	1	6
Couteaux et al. 2019 [16]	1	0	0	1	1	1	4
Dai et al. 2021 [17]	1	0	1	1	1	1	5
Fritz et al. 2020 [18]	1	1	1	1	1	1	6
Hung et al. 2023 [19]	1	1	1	1	1	1	6
Li, Qian et al. 2022 [20]	1	0	1	1	1	1	5
Li, Wang et al. 2022 [21]	1	1	1	1	1	1	6
Luo et al. 2024 [22]	1	0	1	1	1	1	5
Mangone et al. 2023 [23]	1	0	1	1	1	1	5
Pedoia et al. 2019 [24]	1	1	1	1	1	1	6
Qiu et al. 2021 [25]	1	0	0	0	1	1	3
Rizk et al. 2021 [26]	1	1	1	1	1	1	6
Roblot et al. 2019 [27]	1	0	1	1	1	1	5
Shin et al. 2022 [28]	1	0	1	1	1	1	5
Xue et al. 2022 [29]	1	0	0	1	1	1	4
Zhong et al. 2023 [30]	1	1	1	1	1	1	6

Discussion

This study was a systematic review to investigate the diagnostic performance of AI models, such as CNN, or extensions thereof, with regard to meniscus pathology on MRI and how it compared to the performance of orthopedic surgeons and radiologists. Overall, AI was fairly accurate in its ability to detect a meniscus lesion (AUC=0.912). Statistical analysis revealed at least one difference between AI models and physicians with respect to sensitivity and specificity. While the nature of the statistical analysis is not able to determine where this difference lies, upon inspection of the pooled values, it appears promising that AI models performed similarly to physicians with respect to sensitivity and accuracy; however, specificity trended in favor of physicians.

Previous work with regard to AI in the field of diagnostic imaging has shown that on knee and leg radiographs, the specificity of a group of physicians, which included both orthopedic surgeons and radiologists, to detect fractures increased when given the aid of AI versus not [31]. Further, AI has been reported to outperform radiologists on MRI imaging in diagnosing conditions in other fields of medicine [32]. Overall, little work has been done on comparing the performance of AI to physicians, as most studies that do compare AI and physicians prefer to compare physician performance with and without the use of AI assistance. However, a recent systematic review compared the performance of AI models to physicians with respect to musculoskeletal abnormalities on various imaging types, including MRI, and reported mixed results [33]. These results included AI having slightly better accuracy and sensitivity, yet outperformance occurred more often with radiographs and in comparison to physicians outside of orthopedic surgeons and radiologists. With respect to meniscus pathology on MRI imaging, Zhao et al. 2024 have reported on AI’s ability to diagnose, but not locate, meniscus tears based on the status of AI in 2022; however, no comparison was made between the performance of AI and that of orthopedic surgeons and radiologists [7].

It is promising that sensitivity and accuracy appear to be comparable between AI and orthopedic surgeons and radiologists, as it highlights the potential of AI to match physicians’ ability to detect true positives and general overall performance. Doing so is crucial because it decreases the chances of missing the diagnosis of a torn meniscus. As such, one study has shown that AI assistance can increase the sensitivity of radiologists with respect to knee abnormalities, including meniscus lesions, on MRI [13]. High sensitivity of diagnostic tools is vital for early and accurate detection of meniscus tears, as tear pattern, tear extent, and chronicity may impact treatment decisions [34]. Novaretti et al. 2021 reported that the length of small meniscus tears increased after only 100 cycles of knee loading in cadaveric knees, which altered the kinematics of the knee [35]. Moreover, a recent review found that meniscal extrusion and damage, which are common complications of untreated meniscus tears, were associated with progressive cartilage loss and joint replacement [36]. Not to mention, extrusion and further damage beyond the initial tear can lead to increased pain and knee joint instability. Thus, these findings suggest that AI models have the potential to serve as an assistive tool for orthopedic surgeons and radiologists that may provide improved detection of positive cases, ensuring appropriate management and better patient outcomes.

In contrast, the present study identified a trend in favor of orthopedic surgeons and radiologists with regard to specificity, indicating a potentially lower rate of false positives. Decreasing the number of false positives is crucial to clinical management to create an effective treatment plan and avoid unnecessary interventions. While AI demonstrated competent ability to detect positive cases, these findings highlight that AI simultaneously potentially detects false positives at a greater rate than orthopedic surgeons and radiologists, which may be due to the vast number of meniscal variants that can be mistaken for a meniscus tear [37]. Board-certified orthopedic surgeons and radiologists may be better at teasing these variants out than AI, which perhaps might be currently limited in this fashion, given the difference in years of advanced medical training. Thus, while AI may be useful in increasing detection of positive cases, it is important that AI models continue to be refined in order to limit the number of false positives in hopes of avoiding misdiagnoses and inappropriate treatment management.

While meniscus lesions can be diagnosed clinically, it has been reported that when performed by a musculoskeletal physician, gold standard physical examination tests, like McMurray’s and Apley’s, are only 63% and 58% accurate, respectively [38]. Thus, MRIs are and will remain a critical tool in diagnosing meniscus pathology. However, it is important to recognize that the accuracy of radiologist MRI reading was 77.5% and 85.8% for medial and lateral meniscus tears, respectively, when comparing against arthroscopically confirmed positive meniscus cases [5]. Additionally, differences in experience among attending and resident radiologists may impact the ability to correctly identify meniscus lesions [13]. Thus, the results of this review, as well as other studies, suggest that AI algorithms are not wholly statistically inferior to those of physicians and can improve physician performance during imaging interpretation when used in conjunction [14,39].

Limitations

Limitations of the present review include: sample sizes, AI type, and publication bias. As the use of AI for the detection of intra-articular pathologies is novel, few algorithms have been developed, hence leading to a small number of studies included. Thus, more data may be needed to strengthen the results found in this study. It is also important to recognize the difference in sample size among diagnostic metrics for AI and physicians as a potential limitation. Three AI models were pooled and compared to twelve pooled physicians; therefore, results may differ if the two groups were analyzed in a 1:1 ratio. Further, several variations of CNNs, including region-based CNN (R-CNN), deep CNN (DCNN), and 3D-CNN, as well as various extensions of CNNs, such as cascading U-net, UDA, and VIGF, were included in this study, so it is difficult to identify the impact each might have had on AI performance. Further work may be indicated to identify which variation or extension might be most helpful in a clinical setting. Lastly, the results may be skewed by publication bias as only published studies up until June 2024 were included in this review.

Future work

As previously mentioned, future implications include more work to be completed on comparing AI and physician performance. This may provide more confidence in evaluating the incorporation of AI within the clinical setting as an assistive tool to physicians. Future work might also include evaluating AI’s ability to not only detect the presence of a meniscal lesion, but also to identify the type of lesion, the extent of the lesion, and possible recommendations on the indication for surgical intervention.

## Conclusions

The current study suggests that the diagnostic performance of AI algorithms, such as CNN, is comparable to that of physicians. Although there is still room for improvement, AI’s ability to demonstrate high accuracy and sensitivity allows for its potential as a diagnostic assistive tool in a clinical setting to be explored and seriously considered.
